# Integrative Multi-Omics Analysis Reveals Critical Molecular Networks Linking Intestinal-System Diseases to Colorectal Cancer Progression

**DOI:** 10.3390/biomedicines12122656

**Published:** 2024-11-21

**Authors:** Shiliang Ji, Haoran Hu, Ruifang Zhu, Dongkai Guo, Yujing Liu, Yang Yang, Tian Li, Chen Zou, Yiguo Jiang, Guilai Liu

**Affiliations:** 1 Suzhou Research Center of Medical School, Suzhou Hospital, Affiliated Hospital of Medical School, Nanjing University, Suzhou 215163, China; dg21300014@smail.nju.edu.cn (S.J.); 15895571861@163.com (R.Z.); dongkaiguo@126.com (D.G.); 20234133046@stu.suda.edu.cn (Y.L.); yangnj09@163.com (Y.Y.); 2 State Key Laboratory of Pharmaceutical Biotechnology, School of Life Sciences, Nanjing University, Nanjing 210023, China; 231850129@smail.nju.edu.cn; 3 School of Basic Medicine, Tianjin Medical University, Tianjin 300102, China; fmmult@foxmail.com; 4 State Key Laboratory of Natural Medicines, China Pharmaceutical University, Nanjing 211198, China

**Keywords:** bioinformatics, machine learning, intestinal system diseases, CRC, comorbidity

## Abstract

**Background/Objectives**: Colorectal cancer (CRC) frequently co-occurs with intestinal system diseases (ISDs), yet their molecular interplay remains poorly understood. We employed a comprehensive bioinformatics approach to elucidate shared genetic signatures and pathways between CRC and ISDs. **Methods**: We systematically analyzed 12 microarray and RNA-seq datasets encompassing 989 samples across seven ISDs and CRC. Differentially expressed genes (DEGs) were identified using Limma and DESeq2. Functional enrichment analysis was performed using clusterProfiler. Protein–protein interaction networks were constructed via STRING and visualized with Cytoscape to identify hub genes. Clinical significance of shared genes was further assessed through survival analysis and validated by immunohistochemistry staining of 30 paired CRC–normal tissue samples. **Results**: Integrating bioinformatics and machine learning approaches, we uncovered 160 shared DEGs (87 upregulated, 73 downregulated), which predominantly enriched cell metabolism, immune homeostasis, gut–brain communication, and inflammation pathways. Network analysis revealed nine key hub proteins linking CRC and ISDs, with seven upregulated (CD44, MYC, IL17A, CXCL1, FCGR3A, SPP1, and IL1A) and two downregulated (CXCL12 and CCL5). Survival analysis demonstrated the prognostic potential of these shared genes, while immunohistochemistry confirmed their differential expression in CRC tissues. **Conclusions**: Our findings unveil potential biomarkers and therapeutic targets, providing insights into ISD-influenced CRC progression and offering a robust foundation for improved diagnostic and treatment strategies in ISD-associated CRC.

## 1. Introduction

Comorbidity, which was originally defined as “any distinct additional clinical entity that has existed or may occur during the clinical course of a patient who has the index disease under study” [1], has emerged as a critical factor in modern healthcare. Comorbidity is associated with worse health outcomes, more complex clinical management, and increased healthcare costs. This coexistence of multiple diseases within an individual not only poses challenges for patient care, but also raises fundamental questions about underlying etiological connections and their implications for health policy [2]. In the context of cancer, comorbidities can profoundly affect medical care, emotional distress, and mortality rates [3]. Notably, studies have demonstrated that comorbid conditions in breast- and colon-cancer patients are associated with 1.1- to 5.8-fold and 1.2- to 4.8-fold increases in mortality rates, respectively, compared to patients without comorbidities [4]. These findings underscore the critical need for a comprehensive understanding of disease interactions, particularly in complex malignancies such as colorectal cancer (CRC).

The interplay between colorectal cancers and gastrointestinal system (GIS) disorders has been extensively investigated through epidemiological studies [5,6,7,8,9,10], albeit with some conflicting evidence [11,12]. Among GIS disorders, several intestinal system diseases (ISDs) have demonstrated strong associations with colorectal cancer progression [5,6,7,8,9,10]. These ISDs include colorectal conventional adenoma (Tubular/Tubulovillous/Villous adenoma, simplified as TVAD), sessile serrated polyps (SSPs), hyperplastic polyps (HPs), Crohn’s disease (CD), ulcerative colitis (UC), intestinal tuberculosis (ITB), and irritable bowel syndrome (IBS).

CRC development involves multiple precursor lesions, each with distinct molecular and histological features. Conventional adenomas, characterized by nuclear dysplasia, have long been recognized as potential CRC precursors [13,14,15]. Epidemiological studies have consistently shown that patients with advanced adenomas exhibit higher CRC incidence and mortality rates compared to the general population or individuals without polyps [16,17]. Approximately 85% of CRCs are thought to evolve through adenoma-to-cancer sequences associated with specific molecular alterations, including the 5-hydroxymethylcytosine signature in circulating cell-free DNA [18]. SSPs, characterized by BRAF mutations and the CpG island methylator phenotype, represent the major precursor lesions among the remaining 20–30% of CRC cases [19]. These lesions develop cytological dysplasia during colorectal tumorigenesis progression. Recent research has identified the Type II-O plus III/IV pit pattern as a common feature of SSPs with cytological dysplasia in both proximal and distal colon, potentially serving as a hallmark of high-risk serrated lesions [20]. SSPs can develop as primary tumors or evolve from hyperplastic polyps, and their combination of serrated and dysplastic features often leads to rapid progression towards carcinoma [21]. Hyperplastic polyps, the most frequent polyps (80–90%), were traditionally considered benign, but are now recognized as potential precursors, particularly attributed to the difficulty in being distinguished histologically from SSP [22]. Nevertheless, patients with adenoma/polyp subtype had a higher risk of CRC incidence than the general individuals in the largely screening-naïve population. 

The relationship between inflammatory conditions and CRC presents unique challenges in disease progression and treatment. Inflammatory bowel diseases (IBDs), particularly in the case of CD and UC patients, demonstrate distinct pathophysiology from the typical adenoma–carcinoma sequence, and exhibit a higher risk of CRC, accounting for up to 10% of IBD-related deaths [23]. ITB, sharing clinical similarities with CD, presents diagnostic challenges, particularly in regions where both conditions are prevalent. Recent studies have demonstrated that CRC patients with active Mycobacterium tuberculosis infections can safely undergo anti-cancer chemotherapy while receiving appropriate tuberculosis treatment, suggesting potential shared molecular mechanisms [24,25]. 

IBS, a leading functional gastrointestinal disorder affecting over 9% of adults globally [26], presents another dimension to the CRC risk profile [27]. Recent meta-analyses have shown an increased short-term risk of CRC following IBS diagnosis, emphasizing the need for vigilant screening, particularly in younger patients [28]. The clinical overlap between IBS and CRC symptoms, particularly during mild disease activity, can lead to initial misdiagnosis [29]. Given that 24–43% of CRC patients are frail and over half have at least one comorbid condition, the high heterogeneity of CRC sources and undetected molecular signatures presents significant challenges in disease management [30]. This complexity underscores the critical need for effective analytical methods to identify early indicative and prognostic biomarkers.

To achieve early identification and characterization of CRC, we develop a comprehensive framework based on bioinformatics and machine learning models to investigate the role of ISDs in CRC and how they contribute to the occurrence and development of CRC by influencing molecular pathways and genes in CRC. 

## 2. Materials and Methods

### 2.1. Search Strategy and Literature Screening

We systematically searched major databases (PubMed, Web of science) for epidemiological and clinical studies on CRC progression in patients with ISDs published until December 2023, by 2 independent authors (S.J. and H.H.) without language restrictions. We originally studied epidemiological, clinical and gastrointestinal endoscope studies to identify CRC linked to ISDs (i.e., CRCs that are affected by the presence of ISDs). CRC is linked to various gastrointestinal system disorders, among which we selected for study TVAD, SSPs, HP, UC, CD, ITB and IBS. We first obtained some datasets [31,32] that could provide free retrieval. In this work, each disease was searched based on particular criteria (as shown) in the dataset. 

We filtered twelve different microarray and RNA-seq datasets with accession numbers GSE4183, GSE164541, GSE46513, GSE81804, GSE92415, GSE9686, GSE59071, GSE26305 and GSE36701 [33,34,35,36,37,38,39,40,41]. The TVAD dataset (GSE4183 and GSE164541) is the mRNA expression dataset, which consists of biopsy and tissue specimens, which are stored in the RNAlater™ Stabilization Solution at −80 °C [33,40] with 13 control and 20 case samples. The SSPs dataset (GSE46513) is a microarray dataset derived from biopsy samples, which consists of 7 SSPs subjects and 8 control subjects [34]. The HP dataset (GSE81804) is a microarray dataset from 5 patients with Colon polyp with villous component and 5 normal colon mucosa tissues, extracted by NGS [35]. The UC datasets (GSE92415 and GSE9686) are derived from RNA extraction on Affymetrix microarrays derived from the biopsy samples, which consists of 95 UC subjects and 26 control subjects [36,41]. The CD datasets (GSE59071 and GSE26305) are microarray datasets collected from the mucosal biopsies having 10 CD subjects and 13 control samples extracted by Affymetrix Human Gene 1.0 ST Array [37,38]. The ITB dataset (GSE26305) is a microarray dataset from 2 patients with ITB and 2 normal colon mucosa tissues [38]. The IBS dataset (GSE36701) is a microarray dataset derived from biopsy samples, which consists of 87 IBS subjects and 77 healthy volunteers [39]. The CRC datasets (GSE164541 and TCGA) are RNA-seq datasets, obtained from biopsy and tissue specimens, which consists of 652 CRC and 56 adjacent normal tissues [32,40]. To evaluate the patient’s survival for the dysregulated significant genes which overlapped between ISDs and CRC, we acquired clinical and RNA-seq data for CRC from the cBioPortal [42,43]. We used clinical and genetic factors to assess the survival in patients with CRC.

### 2.2. Data Preprocessing and Identification of DEG

Microarrays and RNA-seq datasets based on gene expression analysis are sensitive methods for studying global gene expression and identifying molecular pathways that may be activated in human tissues affected by disease. We can mine these data to identify biomarker genes associated with CRC progression and the survival of cancer patients. We filtered the datasets to select those that show minimum deviation and noise. 

Due to the data generated from different platforms, we use a normalized and Z-score five-point conversion of data preprocessing, in order to avoid complications [44]. For microarray data from GEO, the differentially expressed genes (DEGs) were identified using the Limma R package on normalized count data. For high-throughput sequencing from GEO and TCGA, the DEGs were identified using the DESeq2 R package on normalized count data. The parameters |Log2 fold change| ≥ 1.0 and the False Discovery Rate (FDR) < 0.05 were used as the screening criteria for DEGs. Moreover, the heatmap and volcano plot of DEGs from the databases were constructed using pheatmap and ggplot2 R packages. The Bonferroni, Benjamini–Hochberg and FDR methods were used to adjust *p*-values. Gene-expression dysregulation can be expressed mathematically, as follows:

DEGs =                      Up-regulated  if adj. *p*-value < 0.01 & logFC ≥ 1.0,                       Down-regulated  if adj. *p*-value < 0.01 & logFC ≤ −1.0.

### 2.3. Pathway and Functional Enrichment Analysis

To reveal the potential biological functions and underlying mechanisms of genes and to look over how the factor that contributes to the generation of a trait from the ISDs tissues relates to the expression alterations of the CRC gene, we used the R package “clusterProfiler” to analyze Gene Ontology (GO) and the Kyoto Encyclopedia of Genes and Genes (KEGG) term enrichment of the target genes. GO terms, including biological processes (BPs), cellular components (CCs), molecular functions (MFs), and KEGG pathways with FDR < 0.05, were considered statistically significant.

### 2.4. Identification of Hub Genes

The protein–protein interaction (PPI) network of DEIOSGs was constructed using the STRING, and visualized using the Cytoscape [45]. The highest confidence score of 900 was used as a cutoff value for interaction. The MCODE of the Cytoscape was used to determine the key genes in the PPI network and the setting of topology parameters is described in the previous study [46].

### 2.5. Survival Analysis

CRC is a complex cancer disease caused by genetic and epigenetic abnormalities that affect gene expression. To develop a prognostic model to predict the survival of patients with CRC, we acquired clinical and RNA-seq data for CRC cancer (TCGA, PanCancer Atlas) from the cBioPortal [43] where both clinical and RNA-seq data are available for 647 CRC patients.

We performed the transformation of the RNA-seq data using Z-scores transformation for each gene expression value. Meanwhile, we, determined the altered and normal samples by setting the threshold value as follows:

Z ≥ 2 Overexpress Z ≤ −2 Underexpress 2 < Z > −2 Normal (Unaltered)

To predict the effect of clinical and genetic factors that affect the relative risk of a patient’s survival for biomarker genes that are common to ISDs and the CRC, we applied the standard Cox Proportional Hazards Model for univariate and multivariate analysis [47]. In brief, we identified differentially expressed genes (DEGs) between IBD and CRC samples. These DEGs were then subjected to univariate Cox proportional hazards analysis to assess their individual associations with patient survival. Genes demonstrating significant prognostic value (*p* < 0.05) in the univariate analysis were subsequently evaluated through individual multivariate Cox regression analyses to assess their independent prognostic significance, while accounting for other clinical variables. This systematic approach allowed us to identify robust prognostic biomarkers that maintain their predictive value, independently of other clinical factors.

### 2.6. The ROC Curve Analysis and Expression Analysis

We performed receiver operating characteristic (ROC) curve analysis on each screened hub gene to verify its accuracy. The “pROC” package was used for ROC curve analysis. The hub genes with AUC > 0.8 were deemed useful for disease diagnosis.

### 2.7. Sample Collection and Immunohistochemical Staining Evaluation

In order to verify the expression of 9 hub genes in CRC, the tissues (30 cases of colon cancer tissues with normal paired samples) were purchased from Shanghai Outdo Biotech Co., Ltd. (Shanghai, China), and the research protocols were approved by the Clinical Research Ethics Committee of the hospital.

The 5 μm thick formalin-fixed paraffin-embedded tissue slides were deparaffinized in xylene and rehydrated through an ethanol gradient, ending with a distilled water wash. The sections were repaired in a water bath kettle filled with Tri-EDTA antigen repair buffer and then immersed in 3% H_2_O_2_ for 10 min to block endogenous Peroxidase activity. The sections with antigen retrieval were blocked with 5% bovine serum albumin (BSA) containing 3‰ Triton for 1 h, and primary anti-CD44 (CST, #37259), anti-IL17A (Abcam, ab79056), anti-CXCL1 (Absin, abs120475), anti-SPP1 (Absin, abs110628), anti-FCGR3A (Absin, abs136527), anti-IL1A (Absin, abs113204), anti-c-MYC (GeneTex, GTX103436), anti-CCL5 (CST, #36467) and anti-CXCL12 (CST, #97958) (see Table S3 for details) were incubated overnight at 4 °C. Next, the sections were incubated with the corresponding fluorescent and HRP secondary antibody for 45 min at room temperature. Lastly, all slides were counterstained with DAPI for 5 min and enclosed in ProLong Diamond Antifade Mountant (Thermo Fisher Scientific, Waltham, MA, USA). The specimens were then photographed with a light microscope (BX51; Olympus, Tokyo, Japan) and a Leica TCS SP8 confocal microscope (Leica Microsystems, Mannheim, Germany).

### 2.8. Statistical Analyses

Firstly, the chosen gene-expression datasets and their matrix information were downloaded and converted to Expression Set class for differential gene expression analysis (patients and controls). We created a design model which was then filtered using LIMMA/DESeq2. We identified DEGs through the use of the adjusted *p*-value and absolute log Fold change (logFC) value of the threshold (maximum of 0.01 and at least 1.0, respectively). After comparing them, we obtained all the upregulated and downregulated DEGs. We then constructed upregulated and downregulated disease networks as well as upregulated and downregulated PPI networks, and performed a condensed analysis to identify signaling pathways and ontological pathways. Then, the clinical and genetic factors were prepared for the same patients. Subsequently, the Cox Proportional Hazard Model was used for univariate and multivariate analysis to identify cancer biomarker genes associated with cancer patient survival. Finally, a PL estimator was fitted to construct a survival curve for biomarker DEGs, and we compared our results with those in gold-standard databases and the literature.

## 3. Results

### 3.1. Data Analysis Flowchart

**Figure 1:**
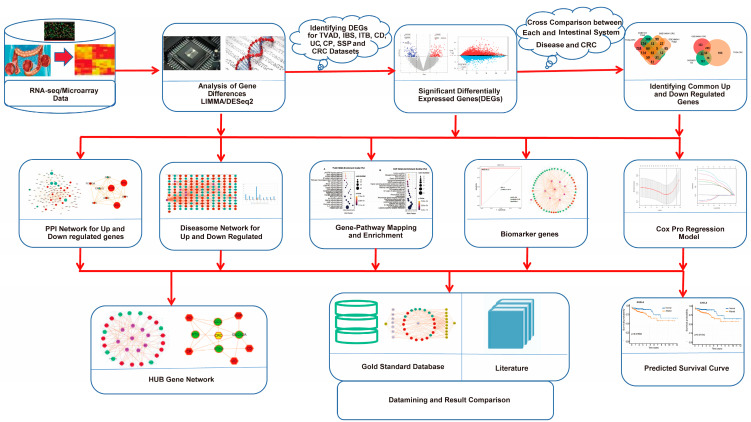
Comprehensive bioinformatics workflow for the analysis of intestinal system diseases and colorectal cancer (CRC). Differentially expressed genes (DEGs) are identified for various intestinal conditions including colorectal conventional adenoma (Tubular/Tubulovillous/Villous adenoma, simplified as TVAD), irritable bowel syndrome (IBS), tuberculosis (TB), Crohn’s disease (CD), ulcerative colitis (UC), colorectal polyps (CPs), sessile serrated polyps (SSPs), and CRC. Significant DEGs undergo cross-comparison between each intestinal system disease and CRC to identify common up- and downregulated genes. Subsequent analyses include (1) protein–protein interaction (PPI) network construction for differentially regulated genes; (2) diseasome network analysis of up- and downregulated genes; (3) gene-pathway mapping and enrichment; (4) identification of potential biomarker genes; and (5) Cox proportional hazards regression modeling. Integration of these analyses yields a hub gene network and facilitates data mining and results comparison against gold-standard databases and the literature.

A sophisticated multistage quantitative framework was designed and developed using an integrated pipeline of bioinformatics and machine learning methodologies to analyze the comorbidity related to ISDs and CRC (Figure 1). This comprehensive approach integrates multiple layers of genomic and clinical data to elucidate the molecular underpinnings of ISD-CRC comorbidity. By leveraging advanced bioinformatics tools and machine learning algorithms, the framework provides a holistic view of the shared genetic architecture and potential prognostic indicators for these interrelated conditions.

### 3.2. Gene Expression Analysis

To elucidate the molecular mechanisms underlying the influence of ISDs on CRC progression, we conducted a comprehensive analysis of gene expression profiles derived from microarray and RNA-sequencing data obtained from the National Center for Biotechnology Information (NCBI) and the National Cancer Institute (NCI) databases. Employing a novel analytical approach, we identified statistically significant differentially expressed genes (DEGs) using stringent criteria (*p* < 0.01 and |log2 fold change| > 1), as summarized in Table 1.

**Figure 2:**
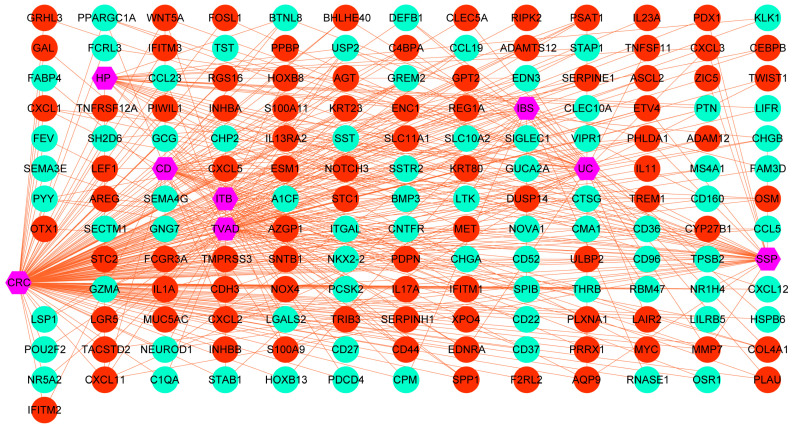
Diseasome network of DEGs shared between intestinal diseases and CRC. Nodes represent individual genes, with red nodes indicating upregulated genes and green nodes representing downregulated genes. Edges between nodes denote known functional or physical interactions between gene products. Central to the network are key disease nodes (shown in purple), including CRC, inflammatory bowel diseases (IBD, UC, CD), irritable bowel syndrome (IBS), and other related conditions such as sessile serrated polyps (SSP) and hyperplastic polyps (HP). These disease nodes serve as hubs, connecting to numerous genes implicated in their pathogenesis.

We then systematically identified overlapping DEGs between ISDs and CRC, which we designated as potential biomarker genes. This was achieved by cross-referencing upregulated and downregulated genes in ISDs with their counterparts in CRC. This analysis revealed distinct sets of biomarker genes for each ISD-CRC pair: 63 marker genes for TVAD and CRC, 52 for CD and CRC, 44 for UC and CRC, 31 for HP and CRC, 40 for SSP and CRC, 40 for ITB and CRC, and 36 for IBS and CRC. To assess the statistical significance and potential diagnostic utility of these biomarker genes, we performed hypergeometric tests and calculated Jaccard indices (Table S1). These analyses provided quantitative measures of the genes’ predictive power for CRC and other ISD-related disorders. To visualize and interpret the complex relationships between CRC and ISDs, we constructed two separate diseasome networks, one for upregulated and another for downregulated genes shared between ISDs and CRC (Figure 2 and Figure S1). These networks offer a systems-level view of the molecular interplay between these conditions. Further characterization of the identified biomarker genes revealed subsets with specific functional roles, including immune-related genes, RNA-binding proteins (RBPs), and transcription factors. The distribution of these functional categories across ISD-CRC biomarker gene sets is detailed in Table S2, providing insights into the regulatory mechanisms potentially driving the observed comorbidities.

### 3.3. Pathway and Functional Association Analysis

**Figure 3:**
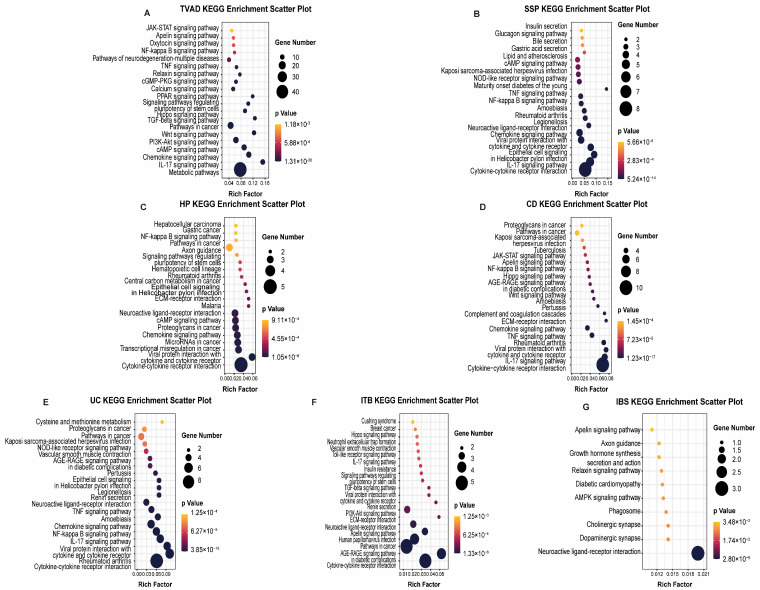
Enriched signaling pathways common to CRC and ISDs. Scatter plots illustrate the enriched KEGG pathways shared between CRC and seven distinct ISDs: (**A**) TVAD, (**B**) SSP, (**C**) HP, (**D**) CD, (**E**) UC, (**F**) ITB and (**G**) IBS. Each panel displays the top enriched pathways based on gene set enrichment analysis. The size of each dot corresponds to the number of genes involved in the pathway, as shown in the legend. The color gradient from black to red represents the statistical significance (*p*-value) of the enrichment, with red indicating higher significance.

To elucidate the molecular mechanisms underlying the comorbidity between Intestinal System Diseases (ISDs) and Colorectal Cancer (CRC), we conducted a comprehensive pathway enrichment analysis using the identified biomarker genes common to both conditions. Utilizing the EnrichR platform and KEGG pathway databases, we systematically explored the signaling pathways activated in each ISD-CRC pair. Our analysis focused on the top 20 statistically significant pathways (adjusted *p*-value < 0.01) for each comparison, as illustrated in Figure 3. Meanwhile, we performed Gene Ontology (GO) term enrichment analysis for the identified biomarker genes to further characterize their functional implications in the context of ISDs and CRC. This analysis provided deeper insights into the biological processes, molecular functions, and cellular components associated with the observed comorbidities.

TVAD and CRC shared enrichment in metabolic pathways, particularly those involved in amino acid and nucleotide metabolism, suggesting alterations in cellular energetics as a common feature. CD and CRC exhibited significant enrichment in inflammatory and immune-related pathways, including cytokine–cytokine receptor interaction and TNF signaling. This highlights the potential role of chronic inflammation in promoting carcinogenesis. UC and CRC are enriched in cytokine–cytokine receptor interaction, NF-κB signaling, and TNF signaling pathways, indicating that persistent inflammatory signaling in UC may drive the progression to CRC. HP and CRC shared enrichment in cytokine–cytokine receptor interaction, transcriptional misregulation, microRNA pathways, chemokine signaling, cAMP signaling, and neuroactive ligand–receptor interactions. This diverse set of pathways indicates a complex interplay of inflammatory, transcriptional, and signaling mechanisms in HP-associated CRC risk. IBS and CRC exhibited shared enrichment in neuroendocrine signaling pathways and gut–brain-axis communication, suggesting a potential role for stress-related factors in carcinogenesis. ITB and CRC showed overlapping activation of pathways related to extracellular matrix remodeling and epithelial–mesenchymal transition, such as focal adhesion and ECM–receptor interaction, indicating potential mechanisms for tissue invasion and metastasis. SSP showed significant overlap in immune-related events and epithelial cell signaling pathways, suggesting that inflammatory processes and alterations in epithelial cell behavior may be key drivers in SSP-related carcinogenesis. 

### 3.4. Protein–Protein Interaction (PPI) Analysis

**Figure 4:**
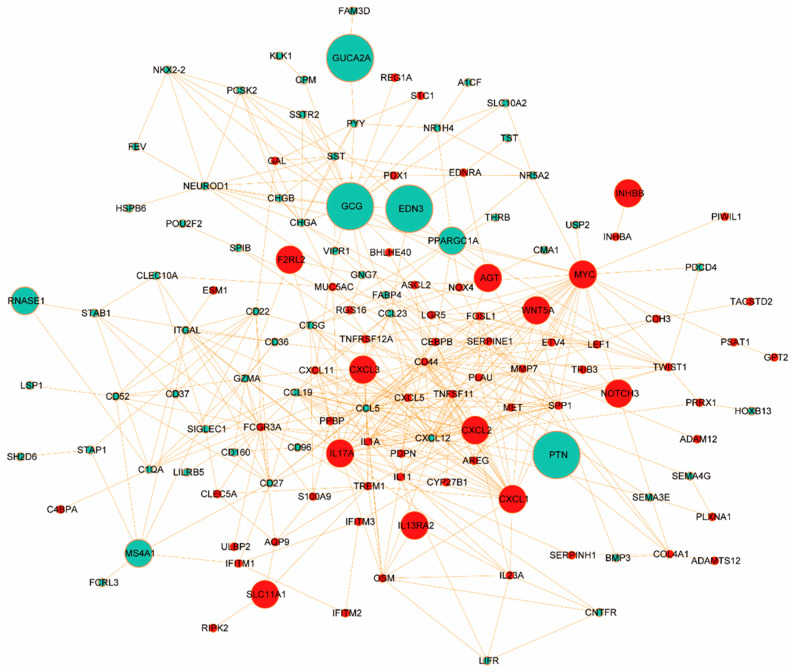
Hub protein network derived from protein–protein interaction analysis of DEGs shared between intestinal system disorders and colorectal cancer. Nodes represent individual proteins, with their size proportional to their degree of connectivity within the network. The color of the nodes indicates the direction of differential expression: red nodes represent upregulated proteins, while green nodes denote downregulated proteins. Prominent hub proteins, such as EDN3, GCG, GUCA2A, PTN, and MYC, are highlighted by their larger node size, indicating their central role in the network and suggesting their potential importance in the molecular pathways linking intestinal disorders and colorectal cancer. Edges between nodes represent known or predicted functional interactions between proteins, with the density of connections illustrating the complex interplay of these molecules.

To further explore the functional interplay among those identified DEGs in ISDs and CRC, we constructed a comprehensive PPI network using the STRING database (https://www.string-db.org/, accessed on 2 December 2023). We applied stringent criteria (confidence score ≥ 0.9 and topological degree > 5) to identify high-confidence interactions, resulting in a network comprising 132 nodes and 456 edges (Figure 4). Protein nodes that did not interact with other proteins were removed. Topological analysis of this network was performed using the MCODE plugin (version 2.0.0) in Cytoscape to detect densely connected regions, which potentially represent functional modules. We identified significant clustering modules based on rigorous criteria (MCODE score > 10 and number of nodes > 20). Subsequently, hub genes were determined using the CytoHubba plugin (version 0.1), focusing on nodes with a degree > 10. Our analysis revealed a distinct set of hub proteins for upregulated and downregulated DEGs, with seven hub proteins (CD44, MYC, IL17A, CXCL1, FCGR3A, SPP1, and IL1A) for upregulated DEGs and two hub proteins (CXCL12 and CCL5) for downregulated DEGs. These hub proteins, illustrated in Figure S2A (MCODE analysis) and Figure S2B (CytoHubba analysis), demonstrate high degrees of connectivity within the network, suggesting their pivotal roles in the molecular pathogenesis of ISD-associated CRC. 

The identified hub proteins encompass a diverse range of functional categories, including cell adhesion molecules (CD44), transcription factors (MYC), inflammatory mediators (IL17A, IL1A), chemokines (CXCL1, CXCL12, CCL5), and immune receptors (FCGR3A). Notably, the presence of both pro-inflammatory (e.g., IL17A) and homeostatic (e.g., CXCL12) factors underscores the complex interplay between chronic inflammation and cancer progression in the intestinal microenvironment. These hub proteins represent potential biomarkers and therapeutic targets for CRC, particularly in the context of pre-existing ISDs. Their central positions in the PPI network suggest they may serve as key mediators in signal-transduction cascades driving CRC progression. Future studies should focus on elucidating the specific mechanisms by which these proteins contribute to the transition from chronic intestinal inflammation to malignancy.

### 3.5. Validation of Hub Proteins with Survival Analysis

We conducted a comprehensive survival analysis using patient gene expression data from the TCGA dataset to elucidate the genes significantly associated with CRC progression and patient survival. Our study focused on genes common to CRC and ISDs of the gastrointestinal system. We employed both univariate and multivariate Cox Proportional Hazard Models, along with the Product-Limit (PL) estimator, to assess the survival function for significant genes in two groups: those with altered and those with unaltered expression. This analysis revealed a set of statistically significant differentially expressed genes (DEGs) common to CRC and the selected ISDs (Table 2). Using a threshold of *p* ≤ 0.05, we identified several genes significantly associated with CRC survival: five genes for TVAD and CRC, eight for CD and CRC, nine for UC and CRC, six for HP and CRC, seven for SSP and CRC, six for ITB and CRC, and one for IBS and CRC. Positive regression coefficients indicated higher hazard ratios and worse prognosis.

**Figure 5:**
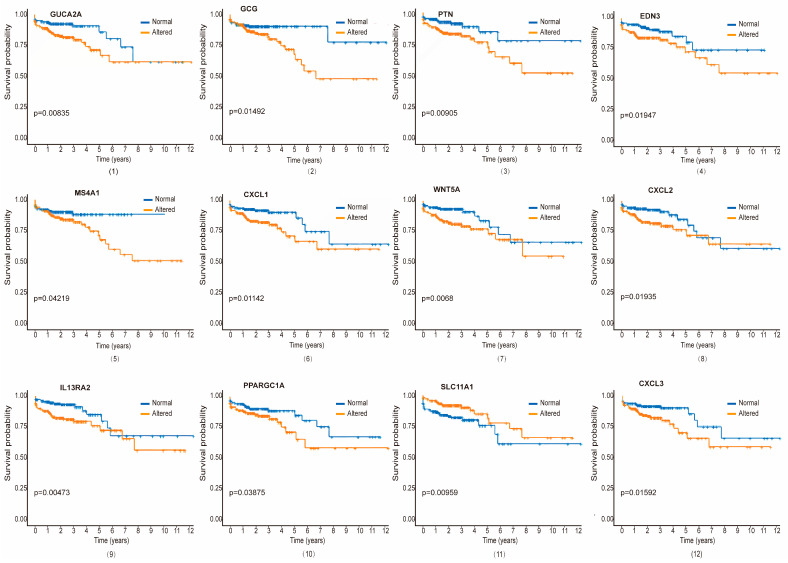
Kaplan–Meier survival curves for key genes shared between ISDs and CRC. Plots present survival analyses for twelve significant genes (GUCA2A, GCG, PTN, EDN3, MS4A1, CXCL1, WNT5A, CXCL2, IL13RA2, PPARGC1A, SLC11A1 and CXCL3) that are commonly dysregulated in both ISDs (including TVAD, CD, UC, HP, SSP, ITB and IBS) and CRC. Each panel displays a Kaplan–Meier plot comparing survival probabilities between patients with altered gene expression (blue lines) and those with normal gene expression (yellow lines) over a 12-year period. The statistical analysis was performed using the Log rank (Mantel–Cox) test.

Survival curves for each significant gene, comparing altered and unaltered expression groups, were generated using the PL estimation function (Figure 5 and Figure S3). As illustrated, we identified 21 genes as the most significant prognostic indicators for CRC patients, with varying associations across different ISDs. Among them, genes such as gene number 1–5 (GUCA2A, GCG, PTN, EDN3, and MS4A1) for TVAD and CRC, 1, 4, 6–11 (GUCA2A, EDN3, CXCL1, WNT5A, CXCL2, IL13RA2, PPARGC1A, and SLC11A1) for CD and CRC, 1, 4, 6–10, 12–13 (GUCA2A, EDN3, CXCL1, WNT5A, CXCL2, IL13RA2, PPARGC1A, CXCL3, and AGT) for UC and CRC, 1–4, 12, 14 (GUCA2A, GCG, PTN, EDN3, CXCL3, and MYC) for HP and CRC, 1–2, 6, 8, 10, 12, 15 (GUCA2A, GCG, CXCL1, CXCL2, PPARGC1A, CXCL3, and ZIC5) for SSP and CRC, 7, 9, 13, 16–18 (WNT5A, IL13RA2, AGT, NOTCH3, OSR1, and INHBB) for ITB and CRC and 19 (RNASE1) for IBS and CRC are associated with survival of the CRC patients. Notably, two genes (F2RL2 and IL17A) were uniquely related to CRC patient survival. In both univariate and multivariate analyses, *p* ≤ 0.05 indicated a significant difference in survival between the genetically altered and unaltered groups. This approach allowed us to determine the joint role of important clinical and genetic factors in CRC patient survival.

### 3.6. Validation Against Gold-Standard Databases and Immunohistochemical Verification

To validate our findings, we compared our results against three gold-standard benchmark databases: dbGaP, OMIM, and Oncomine. We used the EnrichR tool to perform gene set enrichment analysis on the dysregulated DEGs identified in our pipeline, with a *p*-value threshold of <0.05. 

**Figure 6:**
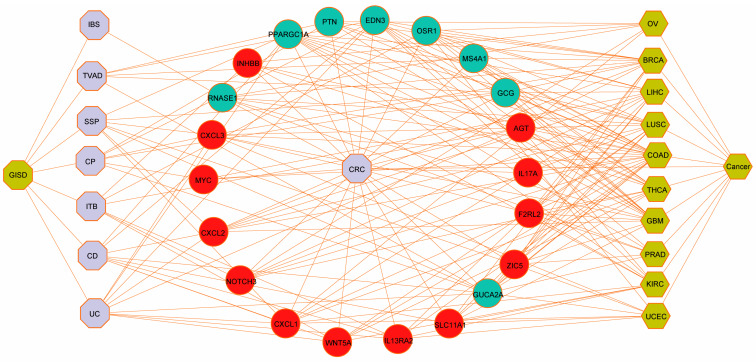
Potential target validation using gold-standard databases. The network diagram illustrates the connections between various diseases and their associated genes, validated using gold-standard databases. The hexagon-shaped light-yellow nodes represent different disease entities. The central purple node labeled ‘CRC’ signifies the broad interplay with intestinal system diseases (shown in the left purple nodes), which includes conditions such as TVAD, CD, UC, HP, SSP, ITB and IBS. The surrounding hexagon-shaped yellow nodes denote specific cancers, including ovarian cancer (OV), breast cancer (BRCA), liver cancer (LIHC), lung squamous-cell carcinoma (LUSC), colon adenocarcinoma (COAD), thyroid carcinoma (THCA), glioblastoma (GBM), prostate cancer (PRAD), renal cell carcinoma (KIRC), and uterine corpus endometrial carcinoma (UCEC). The green and red circular nodes indicate genes associated with these diseases. The network connections, depicted as lines, highlight the intricate relationships between these genes and diseases, providing insights into potential therapeutic targets.

**Figure 7:**
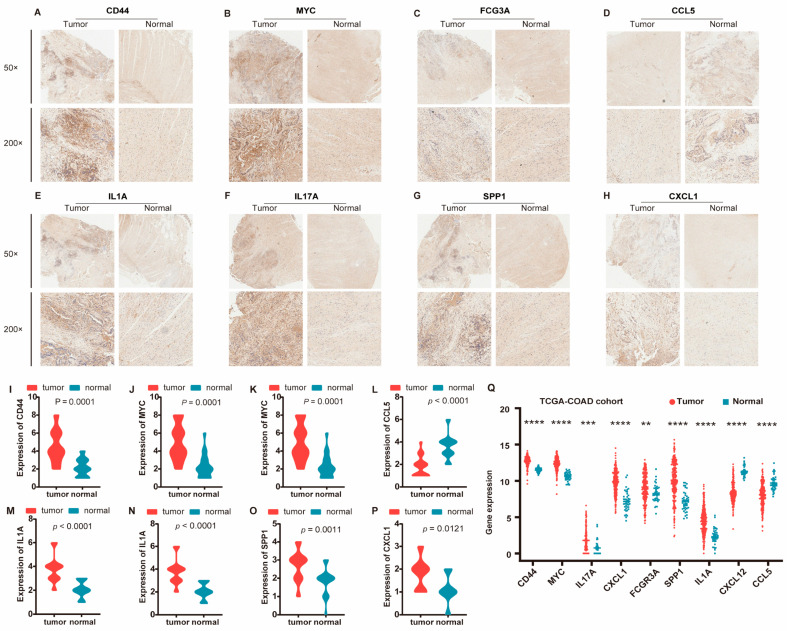
Clinical validation of the identified nine hub genes in colorectal cancer. (**A**–**H**) Immunohistochemical analysis of protein expression for eight genes, including CD44, MYC, FCGR3A, CCL5, IL1A, IL17A, SPP1, and CXCL1, in CRC tumor and adjacent normal tissues. Representative images are shown at 50× and 200× magnifications, illustrating differential expression between tumor and normal samples. (**I**–**P**) Box plots illustrating the expression levels of the eight genes in tumor versus normal tissues. The red color represents tumor samples, while the blue color represents normal samples. Statistical analysis shows significant differences in expression levels for each gene, with *p*-values indicating the extent of differential expression. (**Q**) Expression levels of the nine genes in the TCGA-COAD dataset. The plot shows gene expression (vertical axis) for both tumor (red) and normal (blue) samples, with each dot representing a sample. The genes analyzed are indicated on the horizontal axis. Statistical significance of differential expression is denoted by asterisks, highlighting consistent overexpression in tumor samples compared to normal tissues. ** *p* < 0.01, *** *p* < 0.001, **** *p* < 0.0001.

We constructed a comprehensive disease–gene association network using the list of cancers, as shown in Figure 6, which confirmed that the significant genes we identified in ISDs have known disease associations. This systematic benchmarking against gold-standard data strengthened our confidence in the computational methods employed. For further validation, we performed immunohistochemical analysis on clinical samples, focusing on the nine hub proteins identified by PPI network analysis of DEGs. The immunohistochemical analysis revealed significantly higher expression of CD44, MYC, IL17A, CXCL1, FCGR3A, SPP1, and IL1A in cancer tissues, while CXCL12 and CCL5 showed significantly higher expression in adjacent normal tissues (Figure 7A–H and Figure S4). Gene expression patterns were visualized using the ggplot2 R package (Figure 7I–P). Additionally, we validated these findings using the TCGA-COAD cohort (Figure 7Q).

Overall, our integrated bioinformatics and machine learning approach, coupled with experimental validation, provides strong evidence that ISDs may influence CRC progression. This study not only identifies potential prognostic biomarkers, but also suggests new avenues for therapeutic interventions in CRC.

## 4. Discussion

Our comprehensive study employing integrated bioinformatics and machine learning approaches has unveiled significant molecular and genetic links between ISDs and CRC. In general, results of our analyses indicate that the ISDs share dysregulated genes and molecular pathways and that people with ISD disorders—TVAD, UC, SSPs, HP, CD, ITB and IBS—have a higher chance of developing CRC. By analyzing microarray and RNA-seq gene expression data from both ISDs and CRC, we have provided solid proof that ISDs can interact with CRC at multiple levels. The identification of critical DEGs shared between ISDs and CRC forms the foundation of this interaction. Particularly, our analysis revealed that TVAD shares 63 significant DEGs with CRC, CD shares 52, UC shares 44, HP shares 31, SSP shares 40, ITB shares 40, and IBS shares 36 crucial DEGs with CRC. This substantial genetic overlap not only demonstrates the potential for ISDs to influence CRC development and progression, but also provides a molecular basis for understanding the increased susceptibility to CRC observed in individuals with certain ISDs. The construction of disease–gene association networks further visualizes these intricate connections, offering a clear representation of how ISDs and CRC are intertwined at the genetic level. These networks serve as a powerful tool for identifying key molecular players that may mediate the ISD-CRC relationship and provide targets for future investigations into the mechanisms of this comorbidity.

The functional annotation and enrichment analysis of the dysregulated genes shared between ISDs and CRC have uncovered several key molecular and ontological pathways, providing strong evidence that ISDs have an impact on CRC cancer. These pathways, including those involved in inflammation, immune response, cellular metabolism and gut–brain-axis communication offer mechanistic insights into how ISDs might modulate CRC pathobiology. The identification of hub genes further emphasizes the central role of specific molecular players in mediating the ISD-CRC relationship. These hub genes are implicated in critical signaling pathways that control significant molecular processes in the pathobiology of CRC cancer, such as cell adhesion, inflammation and immune homeostasis. The alteration of these pathways in the context of ISDs suggests that the chronic inflammatory state or other molecular perturbations associated with ISDs may create a microenvironment conducive to CRC development or progression. These findings align with the growing body of evidence linking chronic inflammation to increased cancer risk and provide a molecular framework for understanding this relationship in the specific context of ISDs and CRC [16,17,23].

Our multi-omics analysis, incorporating protein–protein interaction data, further reinforces the impact of ISDs on CRC cancer and provides compelling evidence for the involvement of shared genes in the pathophysiology of CRC cancer. Those shared DEGs between each ISD and CRC underscore the potential for these disorders to influence various aspects of CRC biology. These genes are not merely coincidental overlaps, but represent key molecular nodes through which ISDs may exert their influence on CRC. For instance, genes involved in inflammatory signaling, such as cytokines and their receptors, may contribute to a pro-tumorigenic environment when dysregulated in the context of ISDs. Similarly, alterations in genes controlling cell adhesion or extracellular-matrix remodeling could facilitate tumor invasion and metastasis. The identification of these shared molecular pathways not only enhances our understanding of the ISD-CRC relationship, but also presents potential targets for therapeutic interventions aimed at mitigating the increased CRC risk in ISD patients.

To assess the prognostic significance of the identified biomarkers and further elucidate their role in CRC pathophysiology, we employed the Cox Proportional Hazard Model, a robust statistical method for survival analysis. This approach revealed a total of 50 prognostic genes significantly associated with CRC patient survival. The identification of these genes not only provides potential novel prognostic markers, but also offers insights into the molecular determinants of clinical CRC outcomes in the context of ISD comorbidity. Specifically, we identified five DEGs for TVAD and CRC, eight for CD and CRC, nine for UC and CRC, six for HP and CRC, seven for SSP and CRC, six for ITB and CRC, and one for IBS and CRC as biomarker genes that affect the survival of CRC patients. These findings suggest that the molecular alterations associated with ISDs may not only contribute to CRC development, but also influence disease progression and patient survival. This underscores the importance of considering ISD comorbidity in CRC prognosis and treatment planning, potentially leading to more personalized and effective therapeutic strategies.

The validation of our putative biomarker genes using established databases and independent CRC samples corroborates their relevance to CRC progression and patient survival, further strengthening the evidence for the possible impact of ISD disorders on cancer. The high correlation of these genes with both CRC progression and patient survival suggests that they may serve as valuable tools for risk assessment, prognosis, and treatment selection in CRC patients, particularly those with ISD comorbidities. Moreover, these validated biomarkers may provide important clues for future functional studies aimed at elucidating the precise mechanisms through which ISDs influence CRC biology. Such investigations could lead to the development of novel therapeutic strategies targeting the specific molecular pathways disrupted in the context of ISD-CRC comorbidity, potentially improving outcomes for this high-risk patient population.

However, due to the small sample size from the limited number of studies involved, and different cell types for cross disease or comorbidity analysis, it is possible to miss the genes associated with diseases. Moreover, unpublished gray literature was not searched, although the two searched databases—PubMed, and Web of science—covered the vast majority of relevant journals for the subject. The results in comorbidity biomarker discovery should be further validated in high-quality studies with larger sample sizes, which will further improve the individualized treatment of quality complications in CRC patients. 

## 5. Conclusions

In this work, we explored the use of bioinformatics and machine learning models to integrate and evaluate gene expression, multi-omic, clinical, and molecular data in order to find relationships between disease comorbidity and diseasomes. Our analysis revealed a complex interactome of hub proteins (including immune regulators FCGR3A and IL17A, inflammatory mediators IL1A and CXCL1, adhesion molecules CD44 and SPP1, chemokines CXCL12 and CCL5, and the transcription factor MYC) linking ISD and CRC, underscoring the intricate interplay between immune dysregulation, chronic inflammation, and altered cellular adhesion in disease progression. These findings provide support for the etiologic heterogeneity of colorectal neoplasia and explain why people with intestinal disabilities are more susceptible to developing CRCs. Our suggested methodologies are applicable to identifying a wide range of disease characteristics, particularly those present long before symptoms manifest, and can also aid in improving our comprehension of the intricate pathophysiology of disease-risk phenotypes and the variability of disease comorbidity.

## Figures and Tables

**Table 1:** Statistics of the DEGs for selected intestinal disorders and colon cancer.

Disease Name	GEO Number	Tissues	Platform	Location	Control Samples	Case Samples	Sigt. Genes	UpReg. Genes	DownReg. Genes
TVAD	GSE4183	biopsy samples	Affymetrix Human Genome U133 Plus 2.0 Array	Hungary	8	15	1744	1096	648
TVAD	GSE164541	Triplicate tissue samples	Illumina HiSeq 2500	China	5	5	1121	214	907
CD	GSE26305	biopsy samples	Illumina HumanWG-6 v3.0 expression beadchip	India	2	2	440	228	212
CD	GSE59071	biopsy samples	Affymetrix Human Gene 1.0 ST Array	Belgium	11	8	693	167	526
CRC	TCGA	biopsy samples	High-throughput sequencing	USA Canada	51	647	2141	1169	972
CRC	GSE164541	Triplicate tissue samples	Illumina HiSeq 2500	China	5	5	2082	710	1372
ITB	GSE26305	biopsy samples	Illumina HumanWG-6 v3.0 expression beadchip	India	2	2	877	564	313
UC	GSE92415	biopsy samples	Affymetrix HT HG-U133+ PM Array Plate	Europe	21	87	1187	366	821
UC	GSE9686	biopsy samples	Affymetrix GeneChip Human Genome U133 Plus 2.0 Array	USA	5	8	2013	751	1262
IBS	GSE36701	biopsy samples	Affymetrix Human Genome U133 Plus 2.0 Array	UK	77	87	2137	0	2137
SSPs	GSE46513	biopsy samples	Illumina HiSeq 2000	USA	8	7	746	336	410
HP	GSE81804	biopsy samples	Affymetrix Human Gene 2.0 ST Array	China (Taiwan)	5	5	340	94	246

**Table 2:** The regression results of the Cox model for the DEGs that are common to ISDs and CRC. The parameter legends of the table are Coef for estimated coefficients and HR for hazard.

ISD	Gene Symbol	Univariate Cox				Multivariate Cox			
		HR	HR.95L	HR.95H	*p* Value	HR	HR.95L	HR.95H	*p* Value
TVAD	GUCA2A	1.00002	1.00001	1.00004	0.00500	1.00144	1.00078	1.00210	0.00002
	GCG	1.00009	1.00001	1.00016	0.00207	1.04378	1.02168	1.06636	0.00009
	MS4A1	1.00016	1.00000	1.00032	0.04845	1.05744	1.01616	1.10041	0.03981
	PTN	0.97874	0.96409	0.99361	0.00523	1.00011	1.00006	1.00017	0.00003
	EDN3	1.00075	1.00008	1.00142	0.02923	1.00038	1.00011	1.00064	0.00513
CD	GUCA2A	1.00002	1.00001	1.00004	0.00500	1.00144	1.00078	1.00210	0.00002
	EDN3	1.00075	1.00008	1.00142	0.02923	1.00038	1.00011	1.00064	0.00513
	CXCL1	0.99876	0.99777	0.99974	0.00131	1.01249	1.00631	1.01871	0.00007
	WNT5A	0.99887	0.99816	0.99958	0.00172	1.02524	1.01432	1.03627	0.00000
	CXCL2	0.99921	0.99853	0.99990	0.02483	1.00091	1.00039	1.00143	0.00065
	IL13RA2	1.00003	1.00001	1.00005	0.00057	1.06190	1.03580	1.08866	0.00000
	SLC11A1	1.00046	1.00010	1.00081	0.00119	1.38585	1.18743	1.61742	0.00003
	PPARGC1A	0.99987	0.99975	1.00000	0.04510	1.00033	1.00002	1.00063	0.02638
UC	GUCA2A	1.00002	1.00001	1.00004	0.00500	1.00144	1.00078	1.00210	0.00002
	EDN3	1.00075	1.00008	1.00142	0.02923	1.00038	1.00011	1.00064	0.00513
	CXCL1	0.99876	0.99777	0.99974	0.00131	1.01249	1.00631	1.01871	0.00007
	WNT5A	0.99887	0.99816	0.99958	0.00172	1.02524	1.01432	1.03627	0.00000
	CXCL2	0.99921	0.99853	0.99990	0.02483	1.00091	1.00039	1.00143	0.00065
	IL13RA2	1.00003	1.00001	1.00005	0.00057	1.06190	1.03580	1.08866	0.00000
	PPARGC1A	0.99987	0.99975	1.00000	0.04510	1.00033	1.00002	1.00063	0.02638
	CXCL3	1.00016	1.00002	1.00030	0.00225	1.04923	1.02417	1.07490	0.00010
	AGT	1.00082	1.00043	1.00122	0.00005	1.00710	1.00419	1.01001	0.00000
HP	GUCA2A	1.00002	1.00001	1.00004	0.00500	1.00144	1.00078	1.00210	0.00002
	GCG	1.00009	1.00001	1.00016	0.00207	1.04378	1.02168	1.06636	0.00009
	PTN	0.97874	0.96409	0.99361	0.00523	1.00011	1.00006	1.00017	0.00003
	EDN3	1.00075	1.00008	1.00142	0.02923	1.00038	1.00011	1.00064	0.00513
	CXCL3	1.00016	1.00002	1.00030	0.00225	1.04923	1.02417	1.07490	0.00010
	MYC	1.00086	1.00008	1.00164	0.03002	1.01964	1.00500	1.03449	0.00840
SSP	GUCA2A	1.00002	1.00001	1.00004	0.00500	1.00144	1.00078	1.00210	0.00002
	GCG	1.00009	1.00001	1.00016	0.00207	1.04378	1.02168	1.06636	0.00009
	CXCL1	0.99876	0.99777	0.99974	0.00131	1.01249	1.00631	1.01871	0.00007
	CXCL2	0.99921	0.99853	0.99990	0.02483	1.00091	1.00039	1.00143	0.00065
	PPARGC1A	0.99987	0.99975	1.00000	0.04510	1.00033	1.00002	1.00063	0.02638
	CXCL3	1.00016	1.00002	1.00030	0.00225	1.04923	1.02417	1.07490	0.00010
	ZIC5	1.00016	1.00002	1.00031	0.03053	1.00001	1.00000	1.00002	0.01373
ITB	WNT5A	0.99887	0.99816	0.99958	0.00172	1.02524	1.01432	1.03627	0.00000
	IL13RA2	1.00003	1.00001	1.00005	0.00057	1.06190	1.03580	1.08866	0.00000
	NOTCH3	0.98949	0.98012	0.99895	0.02952	1.00129	1.00038	1.00220	0.00561
	OSR1	1.00007	1.00002	1.00013	0.00140	1.00135	1.00068	1.00202	0.00008
	INHBB	1.00008	1.00005	1.00012	0.00002	1.00767	1.00467	1.01069	0.00000
	AGT	1.00082	1.00043	1.00122	0.00005	1.00710	1.00419	1.01001	0.00000
IBS	RNASE1	1.00824	1.00112	1.01541	0.02315	1.04461	1.01940	1.07044	0.00046

## Data Availability

The data that support the findings of this study are available from the corresponding author upon reasonable request.

## Supplementary Materials

The following supporting information can be downloaded at: https://www.mdpi.com/article/10.3390/biomedicines12122656/s1, Figure S1: Venn diagrams illustrating the overlap of shared DEGs between Intestinal diseases and CRC; Figure S2: Direct network connectivity analysis of key hub genes using different modules of the Cytoscape 3.6.1 software; Figure S3: Survival curve for the most significant DEGs shared between ISD disorders and CRC; Figure S4: Clinical validation of the CXCL12 Gene; Table S1: Hypergeometric test and Jaccard index test for the DEGs genes to establish their role as predictive diagnostic biomarkers for intestinal system diseases; Table S2: The Biomarker genes of intestinal system diseases and CRC were classified according to different functions; Table S3: The antibodies used for IHC in this study.
