# Supplementary material for: Integrative Multi-Omics Analysis Reveals Critical Molecular Networks Linking Intestinal-System Diseases to Colorectal Cancer Progression
Source: Biomedicines. 2024 Nov 21;12(12):2656. doi: 10.3390/biomedicines12122656 (PMC11673540; doi:10.3390/biomedicines12122656)
Supplement: Supplementary file 1 [file biomedicines-12-02656-s001.zip › biomedicines-3302161-supplementary.pdf]

biomedicines

MDPI

Supplemental material

Integrative Multi-omics Analysis Reveals Critical Molecular Networks Linking Intestinal System Diseases to Colorectal Cancer Progression

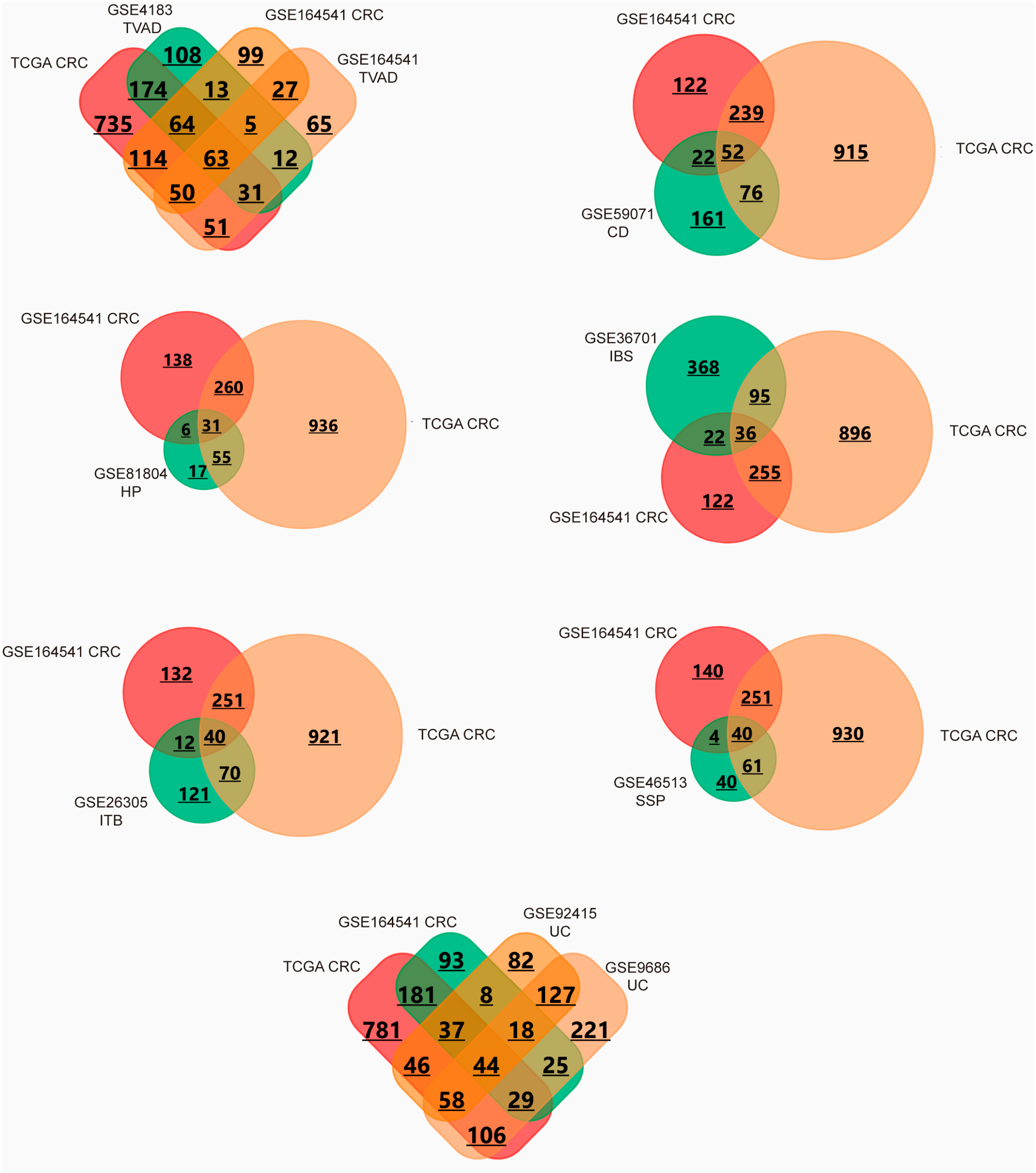

Figure S1. Venn diagrams illustrating the overlap of shared DEGs between Intestinal diseases and CRC.

Each diagram compares DEGs identified in distinct datasets and the numbers within the diagrams denote the quantity of DEGs in each category.

Figure S2. Direct network connectivity analysis of key hub genes using different modules of the Cytoscape software.

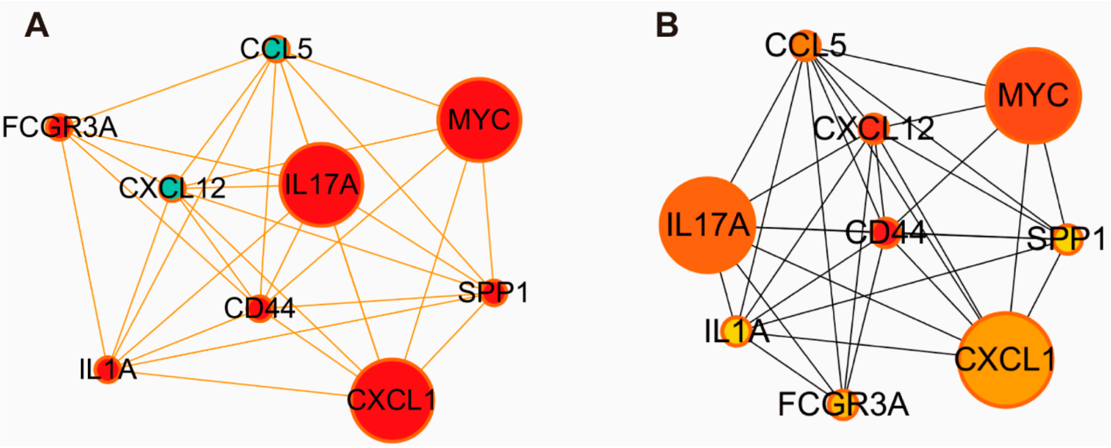

(A) Cytoscape MCODE Module: the network visualization highlights the clustering of hub genes based on the Molecular Complex Detection (MCODE) algorithm. The nodes represent hub genes and the size and color intensity of the nodes correspond to the degree of connectivity, with larger and darker red nodes indicating a higher number of interactions. The edges, represented by orange lines, indicate direct connections between the genes, forming a densely interconnected cluster. (B) Cytoscape CytoHubba Module: The panel displays the network of hub genes using CytoHubba algorithm, which ranks nodes based on their centrality and importance within the network. The nodes are color-coded from yellow to orange based on their hub scores, with brighter colors denoting higher centrality. The edges are depicted with black lines, indicating direct interactions among the genes.

Figure S3. Survival curve for the most significant DEGs shared between ISD disorders and CRC.

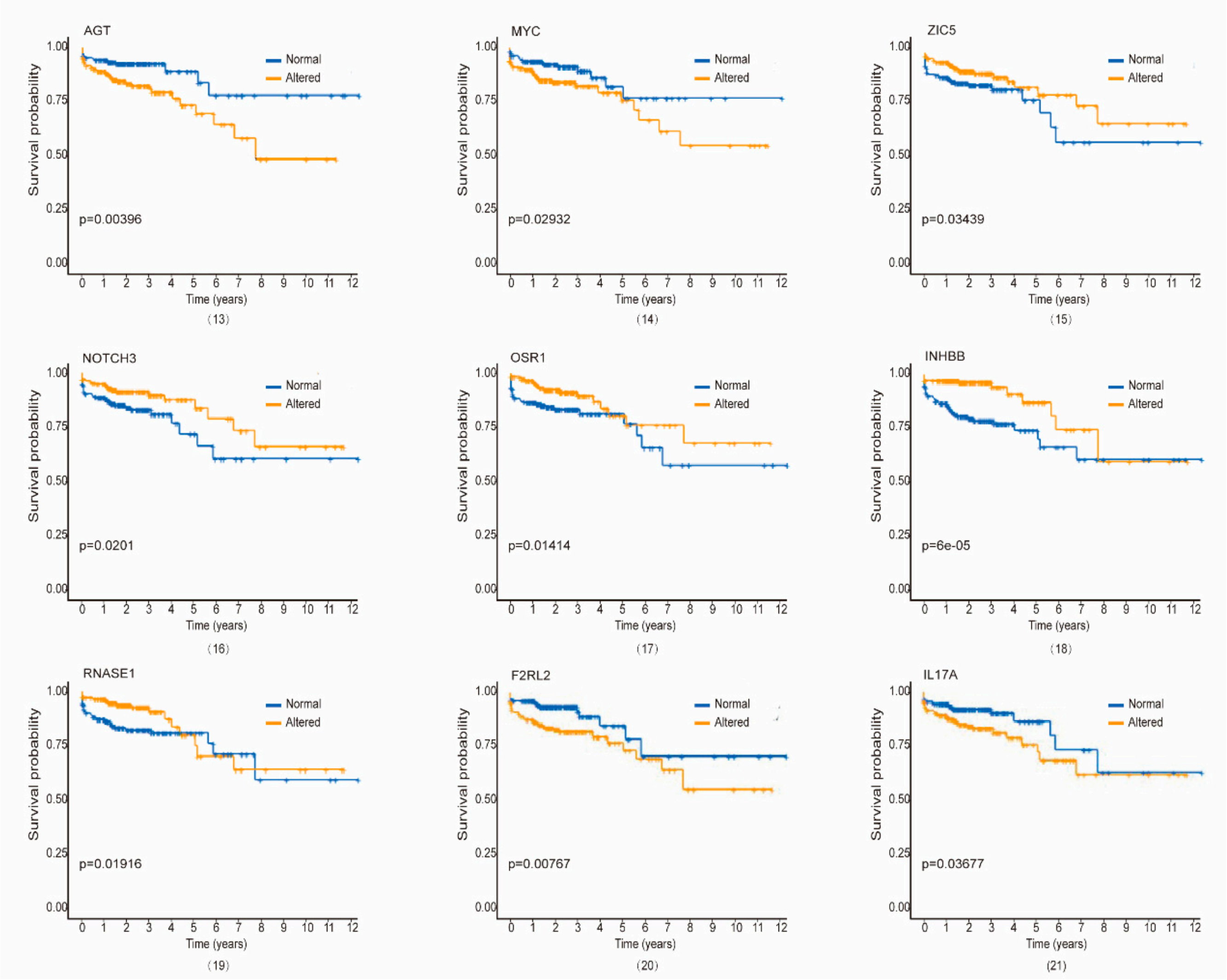

Figure S4. Clinical validation of the CXCL12 Gene.

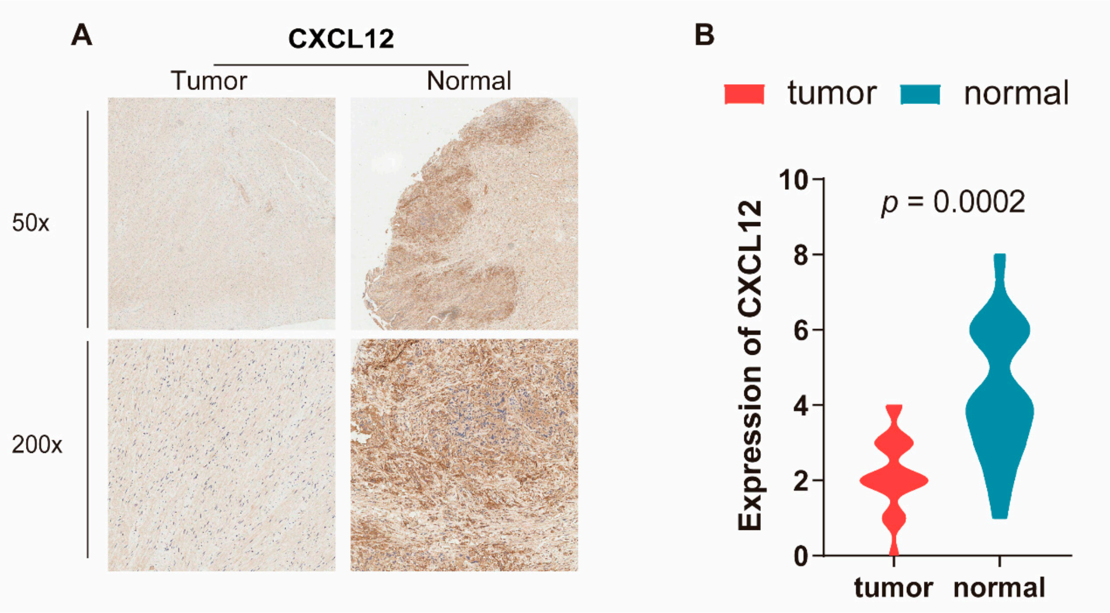

(A) Immunohistochemical analysis of CXCL12 protein expression in CRC and adjacent normal tissues. Representative images were displayed at 50x and 200x magnifications and the staining intensity indicated CXCL12 protein level in tumor tissues and normal tissues. (B) Violin plot comparing the expression levels of the CXCL12 gene between tumor (red) and normal (blue) tissues.

Table S1. Hypergeometric test and Jaccard index test for the DEG's genes to establish their role as predictive diagnostic biomarkers for intestinal system diseases.

Disease Pair	Disease A	Disease B	C (Common between A and B)	Jaccard* index	Hypergeometric p-value
TVAD-CRC	2502	5400	63	1.34E-02	2.119e-06
CD-CRC	411	5400	52	5.42E-03	1.513e-12
UC-CRC	823	5400	44	5.74E-03	9.136e-11
ITB-CRC	877	5400	40	2.40E-02	2.016e-06
IBS-CRC	2134	5400	36	1.44E-02	2.00E-03
SSPs-CRC	741	5400	40	5.34E-02	8.534e-11
HP-CRC	337	5400	31	3.34E-02	2.683e-07

* Jaccard index = C/(A+B-C)

Table S2. The Biomarker genes of intestinal system diseases and CRC were classified according to different functions.

Diseases	Immune gene	RBP	Transcription factors	Total
TVAD	50	4	9	63
CD	36	3	5	44
HP	25	3	3	31
IBS	19	2	3	24
ITB	25	3	4	32
SSP	26	4	9	39
CRC	120	15	26	161

RBP, RNA-binding proteins.

Table S3. The antibodies used for IHC in this study.

Antibody	Cat No.	Manufacturer	Concentration	Application
anti-CD44	#37259	CST	1:200	IHC
anti-IL17A	ab79056	Abcam	1:100	IHC

anti-CXCL1	abs120475	Absin	1:100	IHC
anti-SPP1	abs110628	Absin	1:100	IHC
anti-FCGR3A	abs136527	Absin	1:50	IHC
anti-IL1A	abs113204	Absin	1:100	IHC
anti-c-MYC	GTX103436	GeneTex	1:50	IHC
anti-CCL5	#36467	CST	1:150	IHC
anti-CXCL12	#97958	CST	1:200	IHC
